# From Ethnobotany to Food Innovation: Applications and Functional Potential of *Mashua (Tropaeolum tuberosum)*

**DOI:** 10.3390/foods14234091

**Published:** 2025-11-28

**Authors:** William Vera, Jhonsson Luis Quevedo-Olaya, Hans Minchán-Velayarce, César Samaniego-Rafaele, André Rodríguez-León, Rebeca Salvador-Reyes, Grimaldo Wilfredo Quispe-Santivañez

**Affiliations:** 1 Grupo de Investigación en Desarrollo e Innovación en Industrias Alimentarias (GIDIIA), Universidad Nacional de Frontera, Av. San Hilarión 101, Sullana 20103, Peru; wveraj@unf.edu.pe; 2 Facultad de Ciencias Agrarias, Universidad Nacional de Cajamarca, Av. Atahualpa N° 1050, Cajamarca 06002, Peru; jquevedol@unc.edu.pe (J.L.Q.-O.); arodriguezl@unc.edu.pe (A.R.-L.); 3 Grupo de Investigación en Compuestos Bioactivos a partir de Matrices Alimentarias y Biológicas—COMBALBI, Universidad Nacional de Jaén, Car. Jaén—San Ignacio Km. 24 Sec. Yanayacu, Jaén 06801, Peru; hans.minchan@unj.edu.pe; 4 Escuela Profesional de Ingeniería Agroindustrial, Facultad de Ciencias Aplicadas, Universidad Nacional del Centro del Perú, Tarma 12650, Peru; e_2013101277k@uncp.edu.pe; 5 Facultad de Ingeniería, Universidad Tecnológica del Perú, Lima 150101, Peru; 6 Escuela Profesional de Ingeniería Agroindustrial, Facultad de Ingeniería, Universidad Nacional Autónoma Altoandina de Tarma, Acobamba 120701, Peru

**Keywords:** *Tropaeolum tuberosum*, Andean crop, tuber, nutritional composition, bioactive compounds, functional properties, processing technologies, food innovation, sustainability

## Abstract

*Mashua* (*Tropaeolum tuberosum*), a resilient and underutilized Andean tuber, is a high-potential nutritional and functional food innovation resource. This review surveys information on the nutritional composition, bioactive profile, and functional properties of the tuber based on a comprehensive literature search conducted in the Scopus database. The search strategy employed eight Boolean operators combining the following terms: (“Tropaeolum tuberosum” OR “*mashua*”) AND (“bioactive compounds” OR “functional properties” OR “glucosinolates” OR “antioxidant activity” OR “food processing”). The review included English-language research articles published between 2000 and 2025. Its diverse constituents, such as glucosinolates, phenolics, and anthocyanins, have antioxidant, anti-inflammatory, and antimicrobial activities. Technologies such as freeze-drying, microencapsulation, and 3D printing make it easier to preserve these bioactive components and ensure their use in novel food products. Although *Mashua* has potential, its widespread adoption remains limited by its distinct sensory characteristics and the lack of clinical validation regarding its effect on human health. To extract the full potential of *mashua* as a functional ingredient for the global food industry, consensus evidence exists around the need for standardized analytical methodologies, technological innovation, and sustainable value chain development.

## 1. Introduction

The Andes are one of the major agricultural domestication areas in the world, and they contain abundant sources of Andean roots and tubers, which have been essential to food and nutrient security in high-Andean communities since pre-Inca times [1,2]. In this genetic pool, *mashua* (*Tropaeolum tuberosum* Ruiz & Pavón) is specialized for its broad morphologic and genetic variation and its capacity to adapt to high altitude and marginal soil [3,4].

The dietary characteristics of *mashua* include complex carbohydrates, moderately biologically valuable proteins, dietary fiber, vitamins and minerals, and secondary metabolites, including glucosinolates, anthocyanins, flavonoids, and carotenoids. These compounds exert antioxidant, anti-inflammatory, antimicrobial, and antiproliferative activities, making this tuber a significant nutraceutical and pharmacological source [5,6].

Despite these advantages, *mashua* is an underutilized crop for various reasons, such as low sensory acceptance, no consensus in processing protocols, and no clinical trials for human benefits [7,8]. However, advances in biotechnology, including, but not limited to, metabolomics, micropropagation, and novel processing technologies (such as freeze-drying, microencapsulation, and 3D printing) for this natural resource, allow for new agro-industrial applications [9,10].

**Figure 1:**
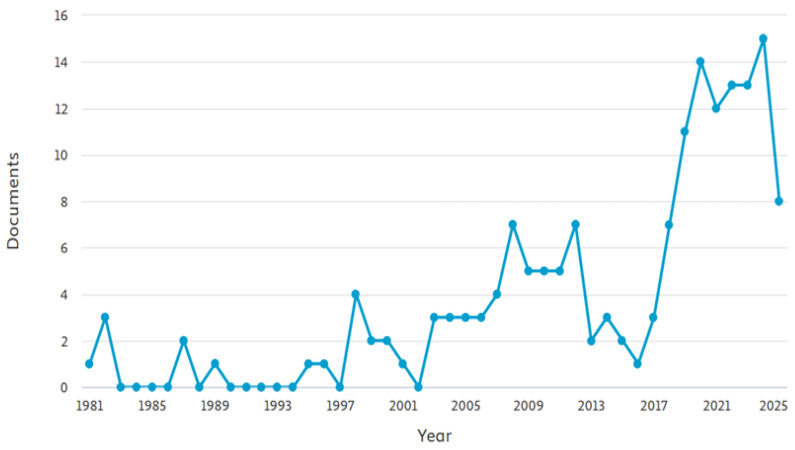
Evolution of the number of Scopus-indexed publications related to *T. tuberosum* (1981–2025).

The number of scientific articles concerning *mashua* from agronomic, biochemical, and technological perspectives has increased significantly over the past years, indicating an increasing academic and industrial interest in this ancestral crop. The archive of indexed data on *mashua* has been steadily growing from the Scopus database (sourced in October 2025), especially since 2018. From 2020 to 2024, the period of the most active publication growth is illustrated in Figure 1, showing a reemergence in the international attention of *mashua* specialists and researchers as a functional and industrially valuable species.

Hence, this study compiles and critically examines research on *mashua*, including morphological, genetic, and ecological diversity, nutrient content and functionalities, processing technologies, and commercial and sustainability considerations. This review aims to help the scientific and technological reevaluation of an ancestral crop that has the potential to become a globally relevant functional ingredient.

## 2. Morphological, Genetic, and Ecophysiological Diversity

### 2.1. Morphological Diversity

**Figure 2:**
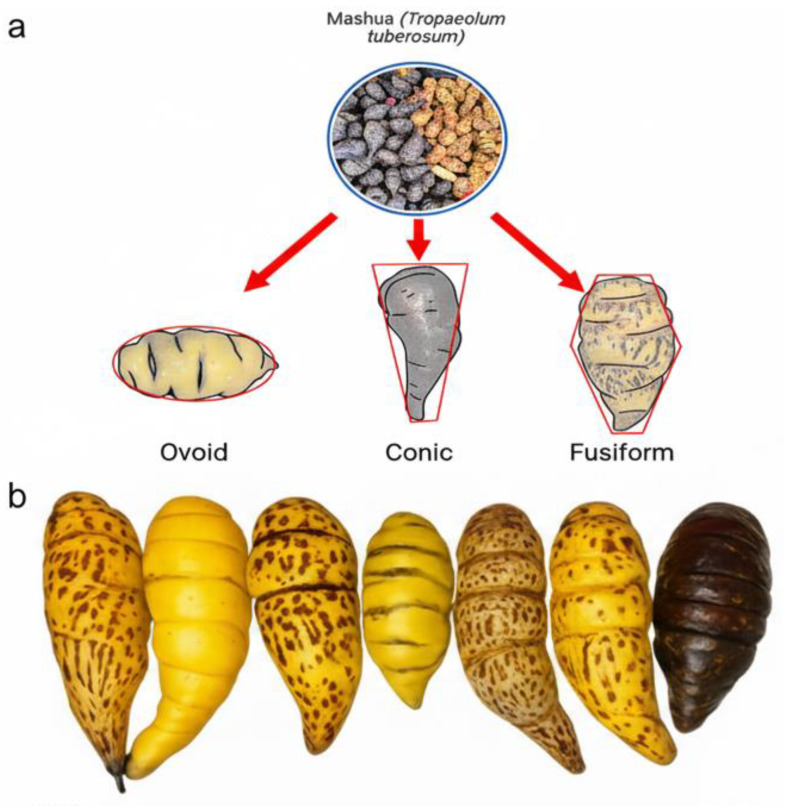
(**a**) Major morphological types of *mashua* tubers: ovoid, conical, and fusiform. Standard outline of normal tuber morphology patterns reported for germplasm morphological characterization. (**b**) Morphological variability and form of *mashua* tubers. In addition to the different colors of tubers, we found a range that reflects the genetic and phenotypic diversity of the species.

*Mashua* exhibits remarkable phenotypic variability across Andean regions and germplasm banks, expressed in tuber shape (conical, fusiform, curved, elliptical, ovoid, and cylindrical) (Figure 2a), skin and pulp coloration (yellow, purple/black, white, pink, or purple) (Figure 2b), and size, with lengths exceeding 0.10 m in certain accessions. These traits are influenced by altitudinal gradients, thermal and water conditions, and empirical cultural selection practices [4,11,12].

In the germplasm bank of the National Institute of Agrarian Innovation (INIA-Cusco, Perú), accessions with differentiated morphotypes have been documented, including tubers with yellow, purple, and black skins, constituting a strategic resource for breeding programs [4]. In Boyacá (Colombia), 25 accessions collected between 2800 and 3200 m.a.s.l. showed broad variability in shape (ovoid, cylindrical, short-thick) and coloration (white, purple, and pink) under a temperate-cold climate of 11–14 °C and precipitation above 1000 mm/year [13].

Elliptical and rounded morphotypes with yellow to purple pulp were detected in Tungurahua (Ecuador) accessions grown from 2600 to 3100 m.a.s.l. [12]. Similarly, the micropropagation of 27 accessions of Apurímac and Ayacucho (Perú) yielded short shapes and dark pigmentation preserved in the experiment under both laboratory and field conditions [14].

This visible diversity, based on appearance, size, and color, represents a long history of domestication and cultural selection. From the practical aspect, this allows for product differentiation strategies (for example, purple tubers in matrices with more robust antioxidant capacity) and adaptability to a particular agroecological setting.

### 2.2. Genetic Diversity and Comparative Context

Although heavily vegetatively propagated, *mashua* exhibits high genetic diversity associated with somatic mutations, farmer selection, and traditional exchange networks. *Mashua* displays a genetic diversity level that differs from that of other Andean tubers. For example, ref. [15], in a comparison with an in situ microcenter of Huánuco (Perú), it was observed that *mashua* possessed higher global genetic diversity rates compared to Oxalis tuberosa (*oca*) and *Ullucus tuberosus* (*ulluco*) using ISSR markers [15]. Whereas *oca* and *ulluco* populations tended to form two disparate genetic groups, *mashua* accessions clustered in a single but highly variable cluster, indicating a greater genetic diversity, providing the possibility for better genetic plasticity in breeding programs [15].

That diversity is heavily shaped by geography. Using data collected from 68 accessions sampled in nine departments in Perú, ref. [11] observed a definite genetic structure based on geographical provenance was observed, which formed three independent genetic groups corresponding to the northern, central, and southeastern parts of the Andes. Similarly, moderate diversity was documented in Colombian accessions with similarity coefficients between 0.54 and 0.81 [13], and high polymorphism was observed in Ecuadorian germplasm [12], indicating that different agroecological niches have unique genetic resources. Importantly, this genetic diversity manifests as functional diversity.

The tuber color dependence (specific gene expression) reflects the tuber’s bioactive potential. The darker genotypes (purple and black) are not only compared morphologically but also as an indication of certain chemotypes possessing considerably increased antioxidant abilities because of the accumulation of anthocyanins and phenolics, with the yellow genotypes more usually connoting different profiles of flavonoids [16,17,18]. Thus, the genetic characterization of *mashua* represents not only a taxonomy issue but also a predictor of its nutraceutical activity.

### 2.3. Ecophysiological Adaptation and Accumulation of Bioactive Substances

**Figure 3:**
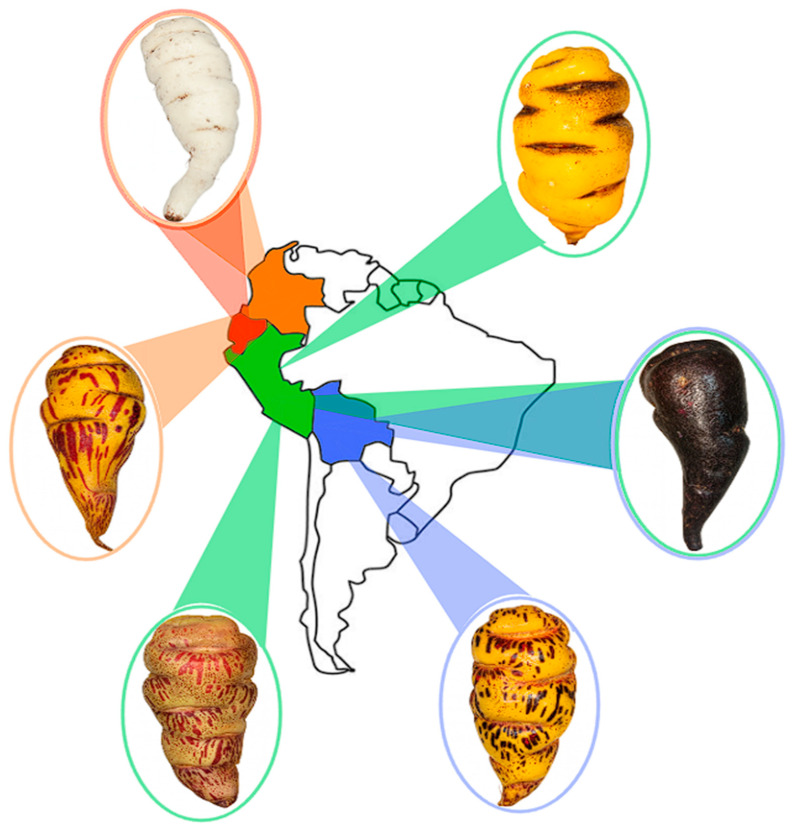
Geographic distribution and phenotypic diversity of *mashua* in South America.

*Mashua* has an extensive habitat in the Andean region’s high-altitude environment (Figure 3) (up to 4300 m.a.s.l.), characterized by low temperatures, poor soil fertility, and high ultraviolet (UV) radiation. Secondary metabolites in this species must be viewed as an evolutionary defensive adaptation to abiotic stresses. Based on the analysis by Aguilar-Galvez et al. [19], postharvest exposure to abiotic stressors, such as sun and cold, elicits stress response systems, including heat shock proteins and osmoprotection compensations. This mechanism is mediated by the accumulation of compounds such as glucosinolates and phenolics, which are antioxidants that counteract the reactive oxygen species (ROS) that are created by environmental stress [19].

Therefore, it is only natural that the Andes climatic conditions are a major driver of bioactivity. Plant phenological cycle and the amount of dry matter accumulated can vary according to photoperiod and temperature due to its plasticity [20]. The reciprocal effect of genotype with environment leads to differential evolution of defense metabolites (glucosinolates [defense against pests and related stress]) from 0.27 to 50.74 µmol/g d.b. (dry basis) depending on the origin of accession (wild or cultivated) [21,22].

It must be noted, then, that the functional potential of *mashua* for food innovation, its antioxidant or antimicrobial properties, is closely connected with its genetic history of adaptation to the harsh Andean ecosystem.

### 2.4. Ecophysiological Diversity

The wide-ranging morphological, genetic, and ecophysiological diversity of *Mashua* provides a strategic advantage against adaptation and valorization (as it is known) by an evolutionary community, but it also presents challenges. It is common in certain plants for many reasons, but most notably in various species. Therefore, the comparison of studies is hampered by disparate methodologies: different molecular markers (i.e., ISSR, AFLP, and SSR) or phenotypic scales prevent researchers from synthesizing results [11,12,15].

Similarly, biochemical characterization using varying analytical techniques (e.g., monitoring spectrophotometry and high-performance liquid chromatography) creates inconsistencies in the quantification of bioactivity [23,24]. The second key element is the disparity in standardization to report results. Although some studies present concentration on a fresh basis, others mention a d.b., which may be misinterpreted if information is not normalized accordingly. This manuscript uses the d.b. and International System of Units (SI) as the norm to ensure comparability.

Finally, while diversity is a type of “biological insurance” for global warming, the agro-industrial exploitation still faces a limitation in the lack of a systematic protocol that addresses how genetic diversity might be coupled with technical characteristics (e.g., antioxidant capacity, texture in processed food, pigment stability) as part of agro-industrial exploitation. Molecular biology, metabolomics, and applied ecophysiology tools should be further integrated in future studies, in combination with studies on technology quality and sensory acceptance [20,21,25]. This strategy will allow for an in-depth characterization of biodiversity and food innovation.

## 3. Nutritional Composition and Bioactive Compounds

### 3.1. Macronutrients

#### 3.1.1. Carbohydrates

*Mashua* contains a significant amount of carbohydrates (56.9–79.5 g/100 g d.b.; starch as the dominant component) [26,27]. Starch content varied between 35.5 and 79.5 g/100 g d.b., indicating genotypic and agroecological variability. In contrast, the starch content in yellow *mashua* was 22.2 g/100 g d.b., which is an intermediate value between that of yellow *oca* (28.1 g/100 g d.b.) and much lower than that of *olluco* (40.1 g/100 g d.b.) grown in Huancavelica, Perú [28]. This distinction is significant for high-content technologies that favor textural characters.

The total sugar content ranges between 6.8 and 55.2 g/100 g d.b., with high percentages of fructose, glucose, and sucrose compared with the potato content [12,29]. The purple genotype produced in Collao, Puno showed 53.32 g carbohydrates/100 g d.b., whereas the same genotype in other areas of Puno showed 70.73 g/100 g d.b., indicating edaphoclimatic influence [30]. The total carbohydrate content (56.9–79.5 g/100 g d.b.) is comparable to *oca* (73.79–76.98 g/100 g d.b.) and *olluco* (74.40–77.50 g/100 g d.b.) under Ecuadorian conditions [9]. In addition, fructooligosaccharide (ketose, nystose) and glucosinolate glycosides with potential as prebiotic ingredients that promote intestinal health by selective stimulation of beneficial microbiota have been reported [31].

The energy value is approximately 1752 kJ/100 g d.b. (419–464 kcal/100 g d.b.), which is positively correlated with the starch content [12,25]. Dietary fiber content ranges from 4.2 to 9.8 g/100 g d.b., indicating that *mashua* is also a moderate source like *oca* (5.78–6.2 g/100 g d.b.) and *olluco* (5.93–6.36 g/100 g d.b.) [30,32]. The inhibitory activity of *mashua* extracts against pancreatic α-amylase and intestinal α-glucosidases (sucrase and maltase) was demonstrated with IC_50_ values of 2.1, 0.8, and 1.2 mg/mL, respectively, indicating a potential for carbohydrate digestion modulation, but moderately less effective than that of acarbose [33].

#### 3.1.2. Proteins

Proteins, the second major component of *mashua*, are additional sources to the complex carbohydrate profile, with significantly higher average values than carbohydrates (ranging between 7.5 and 18.25 g/100 g d.b.) [12,29,32]. The Poza Rondador accession from Chimborazo, Ecuador (2865 m.a.s.l.) reaches a peak value (18.25 g/100 g d.b.), which is related to the long cycle (282 days) and high water demands, especially Kc 1.1, which favors higher nitrogen deposition [12]. In contrast, the Amarilla accession from Tungurahua exhibited a d.b. of 11.19 g/100 g, an indicator of a reduced cycle (169 days), indicating the direct effect of the length of vegetative growth on protein synthesis [12].

Compared with the other varieties, the purple varieties of Puno are predominantly more active (7.41–11.72 g/100 g d.b.) than the yellow (6.96–9.98 g/100 g d.b.) and yellow-purple varieties (7.15–7.95 g/100 g d.b.), implying a potential relationship between pigmentation and nitrogen accumulation. In comparison, *mashua* is higher than all other Andean tubers: *mashua* 9.21 g/100 g d.b., *olluco* 8.06 g/100 g d.b., *oca* 6.84 g/100 g d.b., and conventional potato 6–8 g/100 g d.b., placing it as the highest protein contributor tuber in the group [30].

Proteomic analyses have found that ribosomal proteins and precursors of aromatic amino acids (phenylalanine and tyrosine) are involved in the biosynthesis of glucosinolates, which have two roles, namely, nutrient contribution and involvement in functional secondary metabolic pathways [19,24]. The essential amino acid profile is satisfactory for lysine (5.4–6.2 g/100 g protein) and leucine (6.8–7.5 g/100 g protein) but contains a relatively low content of sulfur-containing amino acids (methionine and cysteine, 1.2–1.8 g/100 g protein), characteristic for tubers and needs supplementation with sulfur-rich sources to maximize the nutritional protein composition in a protein diet [30,34].

#### 3.1.3. Lipids

The minimal lipid fraction, ranging from 0.77 to 2.8 g/100 g d.b., represents the minor behar component [16]. Puno purple genotypes had values of 4.53–6.66 g/100 g d.b. and yellow genotypes had values between 4.60–7.18 g/100 g d.b., showing no clear pattern between coloration and lipid content [30]. It is similar to *oca* (5.5–5.7 g/100 g d.b.) and *olluco* (5.6–7.2 g/100 g d.b.), indicating that the lipid content emerges more from physiological characteristics of the storage organ relative to genotypic variations [30].

The fatty acid profile showed a predominance of linoleic acid (18:2 n-6, ≈20.8%), followed by palmitic and oleic acids [35,36], with unsaturated fatty acids (≈37%) consistently exceeding saturated fatty acids (≈15%), creating a nutritionally favorable profile [16]. Linoleic acid is effective in the prevention of coronary diseases by inhibiting angiogenesis and decreasing blood cholesterol levels [36]. Phytosterols, which are especially *β*-sitosterol, are involved in cardiovascular defense and provide anti-inflammatory and antidiabetic effects [37]. Polyunsaturated fatty acids (omega-3 and omega-6) directly suppress inflammation by inhibiting the actions of proinflammatory molecules [5,38].

In summary, the macronutrient profile of *mashua* showed a predominance of complex carbohydrates (73.79–85.8 g/100 g d.b.) with starch as the main energy reserve, superior protein content (6.96–18.25 g/100 g d.b.) to other Andean tubers, an adequate essential amino acid profile except for sulfur-containing amino acids, and a minor lipid fraction (0.92–1.67 g/100 g d.b.) with a favorable fatty acid profile (37% unsaturated vs. 15% saturated). Table 1 summarizes the proximal composition.

The observed variability in protein and carbohydrate content represents an opportunity for crop valorization through the selection of accessions such as Poza Rondador for its superior protein content, positioning *mashua* as a strategic resource for dietary diversification and nutritional status improvement in high-Andean populations where protein deficiencies and excessive dependence on cereals constitute food security challenges.

### 3.2. Micronutrients

#### 3.2.1. Minerals

*Mashua* has a varied mineral composition with a calcium content ranging from 34.78 to 53.32 mg/100 g d.b. and a phosphorus content ranging from 139.90 to 191.55 mg/100 g d.b. [12,30]. Iron levels range from 7.41 to 7.74 mg/100 g d.b. across purple genotypes [30]. The richest mineral is potassium (1723.42–2021.15 mg/100 g d.b.), followed by zinc (5.0–27.13 ppm) [12,30]. The Poza Rondador accession exhibited higher phosphorus (0.73%) and potassium (2.3%) contents, whereas the yellow *mashua* variety had the lowest values (0.42% P, 0.99% K) correlated with crop cycle length [12]. Mineral diversity among the purple genotypes from different parts of Puno indicated agroecological effects such as that of calcium, ranging from 37.81 to 53.32 mg/100 g d.b., phosphorus, 139.90 to 191.55 mg/100 g d.b., and iron, ranging from 7.41 to 7.74 mg/100 g d.b., attributed to the composition and altitude differences in soils [30]. Among its classic uses, *mashua* is used to treat anemia and genitourinary diseases in high-Andean populations [42].

However, incomplete mineral characterization (frequently omitted magnesium, sodium, and copper) and limited geographic representativeness of studies restrict systematic identification of superior genotypes. The absence of mineral bioavailability assessments limits the nutritional potential of *mashua* to combat specific micronutrient deficiencies.

#### 3.2.2. Vitamins

Ascorbic acid (vitamin C) concentrations range from 0.53 to 4.46 mg/g d.b., with purple varieties exhibiting higher concentrations (1.21–4.46 mg/g d.b.) compared to yellow genotypes (0.53–1.54 mg/g d.b.), positioning *mashua* above other Andean tubers [30]. Total carotenoids vary widely from 1 to 25 µg *β*-carotene equivalents/g d.b., with yellow varieties presenting the highest *β*-carotene concentrations (18.10–715.95 µg/g d.b.), whereas purple genotypes exhibit minimal contents (5.65 µg/g d.b.), confirming the association between pulp coloration and carotenoid profile [30,43]. Castañeta et al. [32] identified through HPLC-MS/MS the presence of lutein, neoxanthin, and *β*-carotene in three Bolivian cultivars, representing the first structural characterization of individual carotenoids in *Mashua* with provitamin A activity. The total carotenoid content in *mashua* (1–25 µg/g d.b.) significantly exceeds that of commercial potato (0.27–3.43 µg/g) and papaya (4.08 µg/g) but remains lower than that of carrot (90 µg/g) and tomato (56–210 µg/g) [43]. Recent studies have reported ascorbic acid content between 0.65 and 4.51 mg/kg in Peruvian morphotypes, which are low values compared to conventional vitamin C sources [23]. Table 2 summarizes the complete micronutrient profile of *mashua*.

Vitamin characterization suffers from critical methodological heterogeneity (expression of results in mg/g vs. mg/100 g, dry basis vs. fresh matter) that prevents rigorous comparisons between studies. The absence of systematic determinations of B-complex vitamins and the lack of assessments of provitamin carotenoid bioavailability limit the evaluation of the actual nutritional potential of *mashua* as a source of fat-soluble micronutrients for populations with vitamin A deficiencies.

### 3.3. Bioactive Compounds

#### 3.3.1. Glucosinolates

Glucosinolates (GSL) constitute the most distinctive secondary metabolites of *mashua*, with total concentrations ranging from 0.27 to 50.74 µmol/g d.b., differentiating wild chemotypes (frequently >25 µmol/g) from cultivated accessions (rarely >5 µmol/g) [17,21,22]. The main GSLs include glucoaubrietin (4-methoxybenzyl, 96–99% of total, 9.6–163.3 µmol/g d.b.), glucosinalbin (4-hydroxybenzyl, 48.6–239.5 µmol/g d.b.), glucotropaeolin (benzyl, 12.8–183.4 µmol/g d.b.), and glucoalyssin (5-methylsulfinylpentyl, 6.7–190.4 µmol/g d.b.), derived from aromatic and aliphatic amino acids [24]. Comparatively, *mashua* substantially exceeds other edible sources: white cauliflower (*Brassica oleracea* var. *botrytis*) 5.27 µmol/g d.b., white cabbage (*Brassica oleracea* var. *capitata*) 21.44 µmol/g d.b., broccoli (*Brassica oleracea* var. *italica*) 17.26 µmol/g d.b., and maca (*Lepidium meyenii*) 31.4–36.2 µmol/g d.b., positioning it as the edible plant with the highest reported content [17,44].

Antinutritional aspects. GSL hydrolysis by myrosinase releases isothiocyanates (ITC), thiocyanates, nitriles, and oxazolidinones, the formation of which depends on the pH, temperature, and cofactors [22,31]. ITCs generate a bitter pungent flavor that limits sensory acceptability and exhibit antinutritional effects at high concentrations without heat treatment. Although no thyroid effects associated with traditional *mashua* consumption have been reported, thiocyanates interfere with thyroid iodine uptake and may induce goiter in iodine-deficient populations [22]. Acute toxicity assays in mice with ethanolic extracts of *mashua* up to 2000 mg/kg produced no mortality or significant adverse effects, being classified as non-toxic according to the Hodge and Sterner scale, evidencing that properly processed consumption poses no lethal risk [45].

Modulation through processing. Boiling, microwaving, and baking completely disable myrosinase without significantly affecting intact GSLs, although leaching during boiling causes losses due to water solubility [17]. Refrigerated storage (12 °C, 80% RH) increases total GSLs by up to 51.5% by day 6, while solar exposure generates 92% losses by day 15, allowing adjustment of levels according to application [17,44]. Freeze-drying preserves GSLs better than conventional drying, although conversion into flour causes significant glucoaubrietin losses due to thermal sensitivity [44]. Total GSLs were reduced in immersion blanching (3–6 min) by 0.5–39.42%, and microwaving resulted in reductions of 32.96–90.67%, suggesting that moist heat treatments could provide fine-tuning [24]. *Lactobacillus rhamnosus* GG undergoes biotransformation in vitro that accounts for 100% of glucosinalbin and glucotropaeolin metabolism, resulting in less toxic derivatives, an evolving approach for functional processing [31]. This genetic diversity enables the selection of cultivated accessions (<5 µmol/g d.b.) that possess moderate contents that, as a combination of heat treatment, exploit functional advantages and reduce antinutritional features.

Current glucosinolate reduction strategies use genetic selection with heat processing, which has proven to be superior in terms of palatability and safety, but may jeopardize the chemopreventive actions that are the basis of *mashua* functional valorization. Studies are needed to study the retention of bioactivity after processing and set consumption criteria that optimize the tradeoff between function and food safety.

#### 3.3.2. Phenolic Compounds

Total polyphenols range from 299 to 2422 mg GAE/100 g d.b., directly correlating with tuber coloration. Caffeic acid concentrations vary between 4.77 and 267.25 mg/100 g d.b., accompanied by rutin (5.58–9.81 mg/100 g d.b.), chlorogenic acid (5.96–25.43 mg/100 g d.b.), and quercetin (0.11–7.66 mg/100 g d.b.) [16]. Chromatographic analysis by HPLC-DAD/MS revealed specific profiles: purple varieties exhibit predominance of chlorogenic acid, gallocatechin, and epigallocatechin derivatives, while yellow varieties present higher catechin and epicatechin content [46,47]. Flavonoids include flavonols (quercetin-3-rutinoside, isorhamnetin-3-rutinoside) and monomeric flavan-3-ols (epicatechin 9.22 µg/g d.b.) with oligomeric proanthocyanidins that contribute significantly to total antioxidant capacity [47,48]

Purple varieties from Puno presented phenolic contents 8–10 times higher than those of the yellow genotypes (90.06–220.83 vs. 77.48–90.83 mg GAE/100 g fresh weight), confirming that pigmentation determines phenolic accumulation [16]. Accession Tt-23 (purple/purple skin/pulp) exhibited maximum levels of caffeic acid (169.19 mg/100 g d.b.) and rutin (9.81 mg/100 g d.b.) with significantly superior antioxidant activity compared with FRAP (2299.03 mM Fe^2+^/100 g fresh weight) [16,37] and DPPH (68.25 µM TEAC/100 g fresh weight) (*p* < 0.05) [16]. Environmental factors modulate these contents: low temperatures and darkness, influence pigment synthesis, whereas prolonged solar exposure induces degradation through photobleaching and oxidation [43].

Phenolic compound characterization in *mashua* lacks studies evaluating bioaccessibility and metabolism of specific phenolics after gastrointestinal digestion and heat processing, limiting their in vivo bioactivity prediction. In addition, the absence of transcriptomic and proteomic analyses to identify structural genes of the phenylpropanoid pathway and genotype-specific transcription factors restricts the development of molecular genetic improvement strategies aimed at optimizing phenolic profiles for specific nutraceutical applications.

#### 3.3.3. Anthocyanins

Purple varieties possess a complex anthocyanin profile characterized by high-performance liquid chromatography–diode array diffraction/MS with derivatives of delphinidin, cyanidin, and pelargonidin [23,43,48]. C3G concentrations vary from 27–160 mg C3G/100 g d.b. and are strongly associated with color intensity and antioxidant potential [23,43]. The main anthocyanins are delphinidin-3-glucoside, cyanidin-3-glucoside, and acylated derivatives, which have gonzales color stability (which is possible under various pH and temperature conditions) [48].

Freeze-drying retains 75–85% of the original anthocyanin content, whereas conventional hot air drying creates losses of >50% [24,49]. Maltodextrin-gum arabic microencapsulation yielded efficiencies of 35.29–84.31% with antioxidant retention of >75%, making [microencapsulation] a promising technology for stabilizing pigments [10]. The potential of natural colorants in the food, cosmetic, and textile industries are a huge industrial opportunity with the increasing importance of synthetic dyes in general. However, the lack of stability studies (prolonged storage >90 days at least) and acylation profile analysis studies in various genotypes limits the optimization of specific industrial applications.

#### 3.3.4. Other Bioactive Compounds

*Mashua* contains alkamides with anti-inflammatory properties. The compound N-(2-hydroxyethyl)-7Z,10Z,13Z,16Z-docosatetraenamide exhibited IC_50_ for TNF-α of 1.56 µM, while N-oleoyldopamine showed IC_50_ of 3.12 µM, both demonstrating potency comparable to synthetic anti-inflammatories [35]. Triterpenoids and phytosterols, particularly *β*-sitosterol and ergosterol, represent significant components of lipophilic extracts from purple varieties, exhibiting hypolipidemic properties and potential effects on prostate health, partially validating traditional ethnomedicinal uses [36,37]. Macamides (*S*)-*N*-(α-methylbenzyl)-oleamide and linoleamide inhibited BSA-MGO formation between 3.39–8.53% and degradation capacity of advanced glycation end products (AGE-protein crosslink) between 6.58–18.08%, suggesting potential applications in managing diabetic complications [50]. However, the absence of studies on the oral bioavailability and metabolism of these compounds limits the evaluation of their physiological relevance in vivo beyond in vitro models. Table 3 summarizes the main bioactive compounds in *mashua*.

## 4. Functional Properties and Biological Activity

*Mashua* also possesses excellent functional properties owing to its rich bioactive compounds, such as phenolic compounds, glucosinolates, and anthocyanins, which have various biological properties. They are associated with antioxidant, anti-inflammatory, antiproliferative, antimicrobial, and organ-protective activities. *Mashua* also makes it an interesting nutraceutical resource. The main bioactive compounds that were named and their functional properties in the *mashua* are systematically classified in various biological systems, as shown in Table 4.

### 4.1. Antioxidant Activity

*Mashua* is morphologically diverse and mainly provides high concentrations of secondary metabolites with nutraceutical properties. Members of this group include phenolic compounds, glucosinolates, carotenoids, vitamin C, hydroxybenzoic acids, flavanols, tannins, anthocyanins, and isothiocyanates. All of these have antioxidant, diuretic, anti-inflammatory, and antineoplastic activities [16].

According to Zeb [59] and Wang et al. [60], phenolic compounds work as antioxidants by neutralizing radicals through hydrogen- or electron-transfer mechanisms, resulting in phenoxy radicals that remain stable in solution after deprotonation through the Sequential Proton Loss and Electron Transfer (SPLET). These mechanisms combine the hydrogen atom being donated, electronic deactivation, and dissipation of the generated radical.

The presence of hydroxyl groups in ortho or para positions also enables the chelation of transition metals such as Fe^3+^ and Cu^2+^, reducing their participation in Fenton-type reactions and, consequently, the secondary formation of highly reactive hydroxyl radicals [61]. In addition, Wang et al. [60] reported that these compounds (caffeic acid, chlorogenic acid, quercetin, and rutin) modulate redox-sensitive pathways, increase the activity of endogenous antioxidant enzymes, and provide antimicrobial and anti-inflammatory effects, linking their presence in *mashua* to cardiovascular protection, metabolic regulation, and attenuation of systemic oxidative damage.

The purple-fleshed accessions have a higher abundance of polyphenols and flavonoids, which accounts for their improved antioxidant activity. In this context, the Tt-23 accession stands out for its higher content of caffeic acid and rutin, compounds known to have been related to Hydrogen Atom Transfer (HAT), Single Electron Transfer (SET), and SPLET [62]. Aligned with the preliminary statement, the chromatographic profile indicates an abundance of chlorogenic acid, gallic acid, quercetin, catechins, proanthocyanidins, and anthocyanins derived from delphinidin and cyanidin [16,46], which is stabilized by the π-conjugated systems and hydroxyl groups through phenoxy radical resonance. This molecular architecture makes the capacity as a barrier to oxidative chain reactions and prevents the formation of reactive species in aqueous and lipid media apparent [23].

In parallel, *mashua* is rich in aromatic and indolic glucosinolates such as glucosinalbin and glucotropaeolin, which release bioactive isothiocyanates through the action of endogenous or microbial myrosinase during processing or digestion. Mechanistic studies of species with similar glucosinolate–isothiocyanate profiles indicate that these metabolites activate the Nrf2/ARE pathway and inhibit NF-κB. These mechanisms reduce the expression of COX-2, iNOS, and various pro-inflammatory cytokines; this mechanism is also inferred in *mashua* [31]. Although anthocyanins exhibit limited bioaccessibility after digestion, they retain antioxidant activity and induce a moderate inhibition of carbohydrate digestion [33]. This suggests that the tuber’s phenolic compounds and pigments act complementarily within its nutraceutical effect.

The antioxidant profile of *mashua* can be changed by postharvest conditions, particularly in the peel, which explains why prolonged storage can increase phenolic levels and antioxidant capacity in purple *mashua* treated with chlorpropham (CIPC) [63,64]. However, this effect diminishes when inhibitor doses are high, revealing the tuber’s sensitivity to postharvest handling conditions [63].

In biological models, purified extracts protect LDL and erythrocyte membranes against peroxidation: at 5 µM gallic acid equivalents, LDL oxidation is reduced by 29.1–34.8% (TBARS) and 51.8–58.1% (conjugated dienes), while oxidative hemolysis of erythrocytes decreases by 20.8–25.1%, effects mediated by flavan-3-ols, proanthocyanidins, and phenolic acids [51]. The ethyl acetate lipophilic fraction (EaF) shows high antioxidant efficacy in lipid-rich matrices. In sacha inchi oil, doses of 200–600 mg GAE/kg extend the oxidative induction period up to 1.9-fold compared with the control and exceed the effect of 200 ppm BHT, while simultaneously reducing peroxide and p-anisidine values during accelerated storage [58]. In raw pork, 100–200 ppm of EaF kept TBARS values below 0.7 mg MDA/kg after 9 days at 4 °C, compared with 2.48 mg MDA/kg in the control, demonstrating sustained inhibition of muscular lipid oxidation, comparable to or even surpassing that of the synthetic antioxidant [58].

### 4.2. Anti-Inflammatory Activity

The available evidence indicates that *mashua* exhibits a verifiable anti-inflammatory effect in vivo and in vitro through the modulation of the early and late phases of the inflammatory response. In the carrageenan-induced edema model, the hydroalcoholic extract of the pink morphotype inhibited more than 50% of the volume increase at the fifth hour, a period associated with prostaglandin synthesis, suggesting the involvement of COX-dependent pathways under the study conditions [36]. This finding is better explained in the cell cultures examined by Apaza et al. [50], where *mashua* alkamides were reported to reduce TNF-α production with IC_50_ values between 5.3 and 10.06 µM, indicating an early effect on the inflammatory cascade and a probable secondary attenuation of NF-κB–regulated mediators, thereby confirming significant inhibition of key mediators and supporting a dual early–late mechanism [35,57].

Apaza et al. [35] and Apaza et al. [57] isolated macamides and alkamides with IC_50_ values ranging from 1.56 to 10.06 µM against TNF-α and NF-κB and described a synthetic analog capable of inhibiting NF-κB at 0.01 µM and activating the Nrf2 pathway at 0.03 nM. These values fall within ranges equal to or lower than those reported for aspirin (IC_50_: 50–100 µM), although they have distinct selectivity profiles.

Silva-Correa et al. [38] demonstrated that 1% topical formulations of black *mashua* induced faster re-epithelialization from day 6 and showed a clearly superior effect from day 8 onward, demonstrating a more efficient wound-healing process than that observed in the untreated control and in a commercial antibiotic ointment. Histologically, treated animals exhibited greater fibroblast proliferation, denser and better-organized collagen fibers, increased formation of new blood vessels, and a more intact epidermal architecture, which explain the accelerated recovery [65]. These effects are attributed to the high content of phenols and anthocyanins (cyanidin-3-glucoside, delphinidin, and pelargonidin), which inhibit NF-κB translocation, thereby reducing early inflammation and promoting faster tissue repair.

### 4.3. Antiproliferative and Cytotoxic Activities

*Mashua* exhibits consistent antiproliferative activity in various tumor cell models. Fuel et al. [66] demonstrated that ethanolic extracts of black *mashua* inhibit the proliferation of colorectal cancer–associated cell lines (IC_50_ between 19.8 and 27.7 µg/mL). The defining feature of this model is that the extract promotes up to a 21-fold increase in intracellular reactive oxygen species’ production and activated caspases (oxidative stress–dependent apoptosis). In addition, along with 5-fluorouracil, it resulted in a synergistic effect (CI < 1). The tuber metabolites synergize well with the cytotoxic effects of this drug by upregulating apoptosis pathways, which may not be 100% recruited by 5-FU [67].

From another perspective, Apaza Ticona et al. [57] isolated alkamide (*S*)-(-)-*N*-(α-methylbenzyl)-linoleamide from *mashua*. These compounds were found to have significant cytotoxic activity (CC_50_ = 1.26 µM), which may be superior to that of synthetic reference compounds such as dimethyl-enastron. This metabolite exerts its antiproliferative activity by inhibiting Eg5, a kinesin critical in spindle generation during cell division. This inhibition of Eg5 promotes the failure of chromosomal segregation, whereas mitotic stress-dependent apoptotic pathways are induced by blocking Eg5.

However, it appears that the bioactive profile is not uniform among all the alkamides in *mashua*. For example, N-oleoyl-dopamine complex compounds could bind to anti-inflammatory receptors without inducing cytotoxicity (CC_50_ > 100 µM), indicating that the antiproliferative effect is selective and structure-dependent and is not related to the alkamide fraction in absolute terms, but to specific analogs [35].

Conversely, Leiva-Revilla et al. [54] and Vásquez et al. [55] found reversible spermatotoxic effects in rodents treated with *mashua* extracts. Both authors noted a decrease in sperm production and motility with no marked morphological changes or reproductive organ tissue damage. Such effects are correlated with the activity of benzyl glucosinolate (3.7 g/100 g of extract), indicating the ability of glucosinolate-derived isothiocyanates to covalently bind to proteins and alter gene transcription in spermatogenesis [56]. Collectively, these findings suggest that *mashua* has selective cytotoxic effects. These effects are mediated by oxidative stress, mitotic inhibition, and covalent reactivity of isothiocyanates, which can preserve tissue structural integrity and modulate the cell processes driving proliferation.

### 4.4. Antimicrobial Activity

The further characteristic of *mashua* extracts is their well-defined antimicrobial activity against various pathogenic microorganisms. With this, Bayas-Chacha et al. [37] obtained the inhibition zones for *Escherichia coli*, Enterococcus faecalis, and *Candida albicans*, as well as the zones for Salmonella spp. inhibition up to 20 mm. The findings demonstrate a strong effect of this on both Gram-negative and Gram-positive bacteria and yeasts. In an additional manner, Peña-Rojas et al. [68] showed that PTC3 inhibits contaminant microorganisms at concentrations as low as 0.2 ppm without damaging plant growth. This property is consistent with the effects of PTS-3, which can inhibit microorganisms at 0.2 ppm. Such actions are due to the isothiocyanate-like activity of some *mashua* metabolites. Covalent integrity and of microbial proteins, the violation of membrane integrity, and inducing oxidative stress led to a powerful inhibitory effect in the models studied, but without phytotoxicity.

In the phytopathological context, Martín & Higuera [21] documented the complete inhibition of *Phytophthora infestans*; this effect was possible using extracts from the Tt-30 accession. Moreover, it has partial effects against *Fusarium oxysporum* (89.5%) and *Rhizoctonia solani* (61.3%), suggesting considerable potential as an agricultural biofumigant. This activity is particularly relevant because these fungal species cause significant losses in high-Andean crops. On the other hand, Ticona et al. [52] and Aguilar-Gálvez et al. [31] isolated an oxadiazole derivative and a phosphonic ester from black *mashua* that exhibits activity against *C. albicans*, *E. coli*, and *Staphylococcus albus*, compounds that reveal distinctive and uncommon biosynthetic pathways within the genus *Tropaeolum*.

### 4.5. Organ and System Protection Activities

*Mashua* also exhibits neuroprotective potential associated with N-benzyl linoleamide–type alkamides, whose derivatives activate the Nrf2 pathway with high potency (EC_50_ = 15.95–21.7 nM) and, in the case of a highly reactive analog, even at 0.03 nM [57]. This activation suggests the induction of ARE-dependent genes, a central defense mechanism against neuronal oxidative stress. Apaza et al. [57] identified structurally related metabolites. These also stimulate Nrf2 induction, further supporting the concept that *mashua* contains compounds that can interfere with endogenous antioxidant pathways. Nevertheless, no evidence exists that these agents can cross the blood–brain barrier, indicating that their in vivo physiological relevance remains undetermined and requires specific bioavailability studies.

Regarding cardioprotective activity, *mashua* lipophilic fractions exhibit amenable cardiovascular activity. Heinert et al. [36] and Chirinos et al. [58] showed that extracts containing *β*-sitosterol, phytosterols, and triterpenoids inhibit angiogenesis, reduce intestinal cholesterol uptake, and protect against oxidative stress. The actions of phytosterols are to decrease the intestinal uptake of 572 dietary cholesterol, whereas triterpenoids and *β*-sitosterol itself modulate the signaling of endothelial 573, suppress VEGF-dependent angiogenesis, and enhance the antioxidant defenses 574 of the vascular wall [36].

Finally, Chirinos et al. [58] determined that the ethyl acetate fraction (EaF) of *mashua* tubers showed an antioxidant capacity of 200.2 µmol TE/mL and 22.2 mg GAE/mL; these levels are associated with protective activity against oxidative stress–induced liver damage. The considerable number of phenolic compounds and lipophilic metabolites in this fraction promotes the neutralization of free radicals by SPLET mechanisms and metal chelation (Fe^3+^ and Cu^2+^) in the hepatic environment and fosters membrane stability, and reduces degenerative processes due to the accumulation of reactive oxygen species.

Phytochemical comparisons between *mashua* and other Andean tubers show that their functional spectrum is comparatively narrower, although these crops exhibit relevant bioactivities. Purple sweet potato displays strong antiproliferative activity (IC_50_ = 1.43 µg/mL) and downregulates IL-1α, IL-18, and IL-6 through Bcl-2/Bax modulation and PI3K/AKT inhibition [69], whereas purple potato shows only moderate hyaluronidase inhibition (21.14%) [70]. Yacon stands out mainly for its antifibrotic response, reducing TGF-*β*1, TNF-α and MDA to baseline at 50 µg/mL by regulating SMAD2/3/4 [71]. Similarly, orange sweet potato has a more restricted profile relying almost solely on polyphenols and *β*-carotene to inhibit NO (69.6%), IL-6 (73%), MCP-1 (69%), TNF-α (56%) and PGE-2 (80%) [72].

In contrast, *mashua* exhibits a combination of anthocyanins, phenolics, glucosinolates, isothiocyanates, and alkamides responsible for the simultaneous modulation of TNF-α, activation of Nrf2, ROS-dependent apoptosis, and Eg5 inhibition, mechanisms largely absent or marginal in the other tubers. This more complex phytochemical profile extends its antioxidant, anti-inflammatory, antiproliferative, and organo-protective capabilities.

## 5. Processing Technologies and Product Development

**Figure 4:**
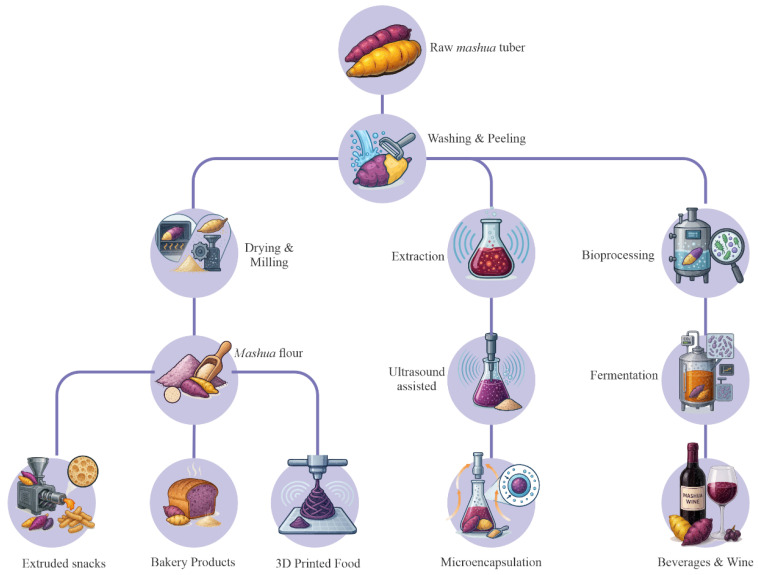
Flowchart of the main technological processing routes and product development in the *Mashua* region.

As detailed in the previous section, the potential of *mashua* as a functional ingredient is intrinsically linked to developing processing technologies that can stabilize and transform it. Consumption of *mashua* in its raw state is restricted not only by its short post-harvest shelf life but also by several essential sensory problems, such as the bitterness and astringency from glucosinolates [17,73]. In addition, many of its important bioactive constituents, including anthocyanins and vitamin C, are labile and easily degraded during storage and conventional thermal processing [23,24,44]. Therefore, the implementation of food science and technology is a fundamental pillar for the revalorization of this tuber. This section critically evaluates the processing operations from primary drying and milling techniques to emerging technologies (UAE, freeze-drying, and extrusion) and biotransformations (fermentation). As illustrated in Figure 4, these are essential for maintaining nutraceutical value, increasing consumer acceptance, and creating novel value-added products [10,49,74,75].

### 5.1. Primary Processing

The *mashua* is preprocessed by washing, peeling, cooking, drying, and milling to produce flour. These actions control the preservation of bioactive materials. For instance, convective drying at 50–60 °C leads to a dramatic reduction in the vitamin C content (up to 45%) and freeze-drying preserves >80% of the initial content [24]. Blanching before drying reduces myrosinase enzymatic activity, thereby attenuating glucosinolate hydrolysis and increasing sensory stability [25].

### 5.2. Emerging Extraction and Processing Technologies

An array of emerging extraction and processing techniques are under investigation to address the extremely short shelf life and improve the industrial performance of *mashua* bioactive compounds. These techniques are not only driven by the necessity of maintaining the integrity of functional components but also to obtain the best yields and stability for the preparation of novel products.

Ultrasound extraction is one of the most effective ways to enrich extracts in the UAE. One of the most relevant studies was the optimization of natural colorants (anthocyanins) and polyphenols extraction from *mashua* by UAE, using Box–Behnken design. The authors assessed the effects of temperature (32–48 °C), ethanol concentration (33–36%), and time (10–20 min). The optimal conditions (48 °C, 33.93% ethanol, and 20 min) allowed for the maximization of the yield of anthocyanins and polyphenols for an extract with significant antioxidant activity (DPPH) [49]. These results confirmed UAE as an effective and scalable approach for extracting the functional components of *mashua* [33].

To enhance the stability of these extracts, microencapsulation through spray drying is a significant tactic. This process was designed for purple *mashua* extracts based on Andean tuber starches modified with octenyl succinic anhydride (OSA) as the encapsulation agent. Using a response surface methodology (CCRD design), the inlet temperature (120–160 °C) and encapsulant proportion (2–12%) were determined. High temperature (160 °C) and relatively low encase proportion (2% of total encapsulants) maximized the EE of anthocyanins, total phenolic content, and solubility, and minimized the water activity and hygroscopicity of products [10].

For dehydration, freeze-drying (lyophilization) is the most reliable method for preserving thermosensitive materials [16,48]. Conventional convective drying (50–60 °C) is more accessible but results in severe loss of vitamin C (about 45%) and serious breakdown of glucosinolates (GSL; reported losses of 38–87%) [24,44]. However, freeze-drying retains >80% of the initial vitamin C and preserves the structure of GSLs, e.g., 4-methoxybenzyl glucosinolate, better than convective drying [24].

Extrusion technology has been developed for the development of second-generation (2G) and third-generation (3G) functional snacks for product development [30]. Experiments have been carried out in laboratory extruders (Brabender and single-screw) with *mashua* flours (e.g., 20%) mixed with corn grits in mixtures as used by experimentally reported cases [74,76]. The addition of *mashua* flour improved the dietary fiber, phenolic compounds, and carotenoid content of the snacks [76]. The *mashua* snacks displayed very few chemical modifications or nutritional losses from the extrusion, confirming the feasibility of retaining their bioactive compounds [74].

3DP is the frontier of food customization. The feasibility of printing purées with *mashua* flour was first evaluated based on pretreatment (cooking or microwave) [9]. The success of 3D printing is strongly rheologically dependent. Formulations must demonstrate solid-like characteristics (G′ > G″). The printed and dimensionally faithful *mashua* (MasC) samples were rated better than the microwave-treated *mashua* (MasM) samples. Cooking induces more denaturation of starch, resulting in retrogradation, which improves the printing viscoelastic properties [9].

Novel advances in bioactive chemistry have revolutionized the stability of *mashua* bioactive compounds. For example, ultrasound extraction (UAE) demonstrated its efficiency, with a total phenolic growth content of 251% and a dry-textured ratio of 1146 mg gallic acid equivalents (GAE)/100 g to DPPH, and antioxidant capacity up to more than 85% [49]. Furthermore, microencapsulation through spray drying has exhibited >75% anthocyanin retention [10], further optimizing their stability against light and oxygen treatment. Similarly, in terms of snack customization, 3D printing approaches to *mashua* flours have established personalized snack textures [9].

### 5.3. Fermented Products

*Mashua* has also been used in lactic and alcoholic fermentation. The production of fermented beverages with *L. plantarum* reached populations of up to 10^8^ colony-forming units (CFU)/mL, with a 25% increase in phenols and a 20% increase in antioxidant activity [77]. Alcoholic beverages are wines with 9.5% *v*/*v* ethanol, good chromatic stability, and an average sensory acceptance of 7.2/9 on the hedonic scale [75].

### 5.4. Bakery and Cereal-Based Products

The addition of BMF to sandwich bread at substitution levels of 5–15% improves the total phenol content (up to 300 mg GAE/100 g d.b.), antioxidant activity (DPPH 43–58%), and reducing capacity (FRAP 6.1–7.3 µmol Fe^2+^/g d.b.), while decreasing the estimated glycemic index (GI) by 12–18% compared to control bread [78]. Consumers showed favorable acceptance of formulations with up to 10% substitution at the sensory level, whereas higher percentages negatively affected texture and color.

Therefore, creating *mashua*-based products is a strategic balance between valuing its functional compounds and mitigating some tech issues. Preservation technologies such as freeze-drying, microencapsulation, and ultrasound-assisted extraction keep bioactive phenols and anthocyanins, but their industrial scalability needs further validation alongside improvements to the sensory acceptance, as products containing intense coloration and bitter glucosinolate-derived notes (e.g., fortified snacks, breads) may experience limited consumer appeal despite moderate acceptance (see Table 5). Although *mashua* has been incorporated into numerous types of food mixtures (e.g., bakery products, fermented beverages, or functional snacks), and it appeals to consumers seeking nutritious and nourishing foods, its commercialization can be aided by following established processes to retain bioactive compounds with a precision that aligns with the taste of the consumer, avoiding perceptual barriers through appropriate ingredient blend, and validating the bioaccessibility of key compounds in final products to ensure health and wellness.

## 6. Commercial Perspectives and Sustainability

The transition of mashua from an ancestral crop to a strategic industrial ingredient is based on its bioactive compounds, including glucosinolates, anthocyanins, and phenolic acids, which confer antioxidant, anti-inflammatory, and antimicrobial properties (described in Section 4 and Section 5). This functional basis enables the development of foods and nutraceuticals aimed at improving human health, translating into differentiated commercial opportunities in specific markets (Table 6).

### 6.1. Segmentation of Emerging Markets

Market for high-quality functional ingredients: Varietal classification enables commercial discrimination, and purple accessions, such as Tt-23 (220.83 ± 0.42 mg GAE/100 g polyphenols; 79.66 ± 0.19 mg CE/100 g flavonoids), are aimed at high market niches, and yellow varieties are aimed at the market (mass) sector [16,26,30]. Visual classification is facilitated by the strong correlation between pigmentation and phenolic content (R^2^ = 0.7), reducing dependence on costly chemical assays in industrial processes. The chromatographic profiles are specific (the dominant chlorogenic acid in purple and catechins in yellow product lines), allowing differentiated product lines to be developed in contrast with undifferentiated products [64]. This biochemistry specialization also reflects the added value in different market sectors.

Natural industrial colorants market: Stable anthocyanin pigments have the potential to substitute synthetic dyes in real time and have proven applications in cosmetology and as traditional dyeing materials in textile production [79]. Their dimensional stability in 3D printing extends their use to personalized foods, thereby widening their commercial channels [9].

Biodegradable packaging markets: *Mashua* starch-based films have satisfactory barrier properties, excellent transparency, and reasonable thermal stability for food packaging, thereby providing a sustainable and commercially viable alternative [80,81].

### 6.2. Technological Scaling Strategies

Commercial extraction standardization: Ultrasonic extraction (20 kHz, 150 W, 15 min, 60% ethanol) provides 251 mg GAE/100 g and has a DPPH of >85%, which is superior in terms of time-energy efficiency to conventional methods [49]. The technology is transferable into industry, thus there is an expected ROI. Maltodextrin–gum arabic (MDGA) microencapsulation (efficacy, 35–80%; antioxidant preservation, >75%) represents an efficient technology toward commercialization and stability for incorporation in a broad range of food matrices [10].

Further differentiation of postharvest optimization: In contrast, more phenolics are produced during longer storage than during rapid breakdown by sunlight, and this necessitates a modified protocol according to the product being marketed [63]. Freeze-drying of bioactive compounds for >60 days outperforms thermal degradation and may provide a strategy for high-end products [26,49].

### 6.3. Value Chain Models

By establishing integrated agro-industrial chains, Andean vertical integration takes advantage of rusticity and altitudinal adaptation for a competitive edge and plays a major role in regional food security and export possibility [79]. SIT-RITA^®^ micropropagation of the products promotes sanitary quality and genetic traceability for international markets [83]. Inter-agroecological variability (2.73–6.825 mg/g DW polyphenols) leads to site-specific optimization with potential of origin-designation, thus generating territorial added value in markets where the authenticity of the product is a need [16,33].

### 6.4. Industrially Scalable Products

The potential of *Mashua* for the industry has been affirmed with the validation of several high-volume products. Snack extrusions (within desirable parameters, 71–84%, density 0.15–0.23 g/cm^3^) show scalability, and functional flours demonstrate strong consumer acceptability (>80%) and validated antimicrobial performance against S. aureus. In addition, fermented beverages provide certain valuable properties, such as 9.5% *v*/*v* alcohol content, 7.2/9 acceptance score, and 60-day stability [74,75,78]. The vacuum-fried chips look particularly promising because of their low moisture concentration (1.63%) and significant fat decrease compared with their conventional counterparts (50%) and thus meet established vegetable product standards (INEN < 40% fat), which can make them stand out in comparison to conventional products [25].

Meanwhile, new specialized products are showing a technological divergence: from one extreme, the extreme precision of individually made 3D printing is at the opposite of the cost scale of large-scale microencapsulation. The current bifurcation also points to the probable future split of premium and more personalized nutraceuticals versus high-volume commodity ingredients [23,74,84].

Therefore, *Mashua* is poised to leverage as a key asset that aligns with a global shift in consumer value in functional, sustainable, and culturally unique foods. Its commercialization, however, would not be automatic; it requires a solid, careful strategy that carefully synthesizes powerful scientific innovation with very selective clinical validation and rigorous quality standardization with an attitude of the inclusion of market developers in the way of respecting the product’s original place of origin.

## 7. Current Challenges and Future Research Directions

### 7.1. Current Challenges and Limitations

In recent years, considerable attention has been paid by the scientific literature to the optimization of several extraction, preservation, and processing techniques used for *mashua* to assure the stability and bioavailability of *mashua*’s major functional compounds (glucosinolates, phenols, anthocyanins, and bioactive lipids) when integrated in food matrices (Table 7) [24,31,73]. Therefore, novel and advanced technologies from controlled extrusion, 3D printing, and non-thermal treatments have emerged as important avenues to preserve that functionality and significantly extend the shelf life of produced products [9,26,74].

From a biotechnology perspective, metabolomics and thorough phytochemical characterization have emerged as strong tools. This advanced profiling is essential for the efficient selection of ecotypes appropriate for different applications (nutraceutical, industrial, or pharmacological) and for the development of targeted bioprocesses that can be used to produce very specific functional agents [23,84]. Similarly, approaches, such as micropropagation in SIT-RITA^®^, provide a solid and cost-effective alternative source of disease-free seeds, saving essential genetic diversity, and enabling the reliable scaling-up of productive Andean germplasm production [83].

From a pharmacological perspective, *mashua* has clearly shown potential as an easily available source of therapeutic molecules with considerable anti-inflammatory, antioxidant, antimicrobial, neuroprotective, and anticancer activities. Newer studies indicate that glucosinolates and bioactive lipids could become synthetic analogs targeting biomedicine and the nutraceutical industry [38,52,56,57]. Nevertheless, its safety and efficacy must be confirmed in vivo, clinically, and by toxicological evaluations [35,85].

Finally, and at the highest social and cultural level, the significance of public policies and promotion strategies that value Andean foods is seen, favoring their inclusion in food and nutrition security programs and economic development in rural areas [7,75,86].

### 7.2. Challenges in Developing Functional Products

*Mashua* has an attractive profile for the development of functional foods, characterized by its high protein content (approximately 18%) and high concentration of phenolic compounds and anthocyanins, which give it an antioxidant capacity that exceeds that of other Andean tubers, such as *olluco*, *oca*, and native potatoes [16,30]. Its distinctive value lies in its glucosinolates, which are hydrolyzed into health-promoting isothiocyanates, but simultaneously impart a characteristic pungency that needs to be mitigated [73].

Advances in research and development have led to the development of promising prototypes, such as gluten-free flours, extruded snacks with higher bioactive content, pigmented yogurts, and functional beverages [49,74,75]. These innovations seek to integrate *mashua* into food matrices, where it increases antioxidant capacity and provides natural coloring, thereby reducing the dependence on synthetic additives.

However, substantial commercial market challenges persist, requiring urgent technological intervention [11,75,76]. The same glucosinolates driving the functional benefits [19,37] of *mashua* also represent multiple barriers to market entry: these compounds, as pungent and bitter compounds, generate substantial sensory disturbances that directly affect consumer acceptance [11,22] and exhibit antinutritional properties. These effects include the possibility of goitrogenic activity that can affect thyroid function and iodine metabolism as well as lower protein digestibility and mineral bioavailability. Thus, research into more advanced pretreatment technologies is urgently needed [24] to overcome these problems while still retaining some of the advantages of isothiocyanates released during glucosinolate hydrolysis. New approaches in this field include genetic strategies for glucosinolate profile modulation, optimized thermal processes (conventional and microwave), fermentative control, and selective extraction techniques [88,89,90].

Although *mashua* is an interesting and valuable protein–bioactive mix, the future development of *mashua*-based functional foods rests on striking the optimal level of bioactivity-palatability. The planned application of targeted processing technologies is a key next step to fully utilize *mashua* as a sustainable and health-aware ingredient in the entire food product range worldwide.

### 7.3. Research Trends

**Figure 5:**
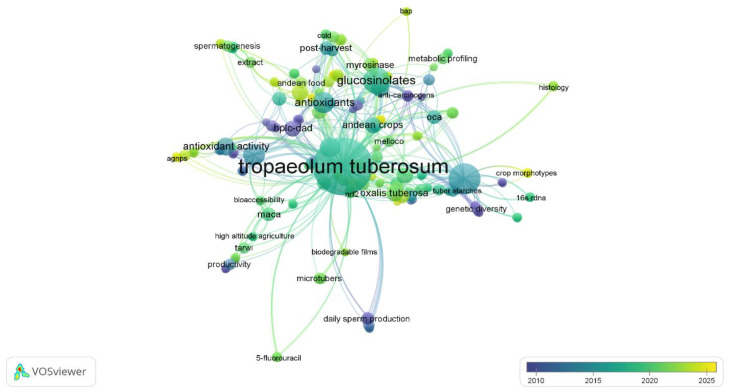
Bibliometric analysis of terms associated with *mashua* in the scientific literature (2010–2025). Network visualization (VOSviewer v.1.6.20) highlighting concepts such as “glucosinolates,” “antioxidants,” “genetic diversity,” and “food processing” represent the main research lines.

More recently, the literature has been highlighting the importance of seeking research trends on extraction and has focused on increasing extraction efficiency in recent years. Sustained growth of scientific production of *mashua* has been observed in recent years (and it remained in its infancy compared with other Andean tubers). As shown in Figure 5, the keyword co-occurrence analysis revealed *mashua* to be the network’s central node (52 occurrences; total link strength: 243) and highly related terms, including glucosinolates, antioxidant activity, phenolic compounds, and HPLC. This articulation captures the current trend toward characterizing their bioactive factors and the investigation of their nutraceutical potential (especially in pigmented varieties that are highly enriched in isothiocyanates, anthocyanins, and flavonoids) [23,32].

Incisive advancements in instrumental techniques, such as high-performance liquid chromatography (HPLC), spectrophotometry, and mass spectrometry, have enabled a more fine-grained characterization of metabolites with antioxidant, anti-inflammatory, and antiproliferative activities. These results are also associated with the modulation of key molecular pathways, such as NF-κB, Nrf2, and TNF-α, which have important applications in the prevention of chronic non-communicable diseases [16,30,35,57]. Similarly, bioaccessibility research and investigation of phenolic compounds for food and pharmaceutical preparation have introduced functional foods and natural supplements.

In the temporal dimension, bibliometric analysis shows the recent emergence of trends such as bioaccessibility, biodegradable films, extract, and 3D printing, reflecting a transition toward innovative technological and therapeutic applications [31]. These lines point to the development of biomaterials, biodegradable films with antioxidant activity, and controlled-release systems for bioactive compounds. The integration of *mashua* into 3D printing platforms for functional foods has also emerged as a promising field for diversifying its applications in the food industry [9,27,76].

**Figure 6:**
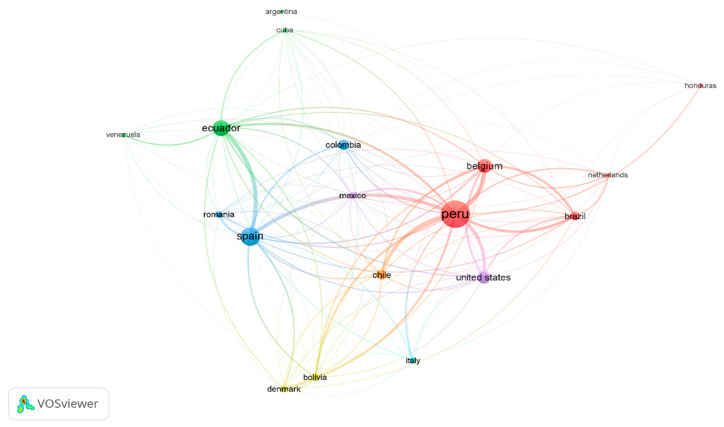
International collaboration in *mashua* studies Bibliometric map showing scientific cooperation among countries, with Perú, Belgium, Spain, Ecuador, and Brazil as the main nodes.

In contrast, the bibliographic coupling analysis (Figure 6 indicates that Perú is the leader in scientific *mashua* production (48 documents; 1047 citations), followed by Belgium, Spain, the United States, Brazil, and Ecuador. This position is due to the country’s role as an Andean producer and diversity center, where germplasm supply and scientific research in biodiversity, phytochemistry, and technology are emphasized [64,84]. Perú is represented as a node articulating an active international network with excellent cooperation with Belgium, Spain, and Brazil in phytochemistry, biodiversity, and food security. The ecosystem evidence that *mashua* is not only a local agro-resource but also contributes strategically to global interdisciplinary research.

Some key gaps remain to be filled, such as validating the bioavailability of active metabolites through in vivo and clinical studies and standardizing extraction and purifying procedures for bioactive compounds [24,74,76]. The extension of these capabilities to new techniques by harnessing molecular biology in combination with functional genomics tools will result in improved *mashua* accessions with better nutraceutical profiles, advanced metabolomics, and CRISPR-based gene editing.

Lastly, enhancing regional cooperation between Andean *mashua*-producing countries, which exhibit more scientific and technological capabilities, is important. Further expansion of germplasm banks, exchange of protocols, and adoption of suitable technologies will consolidate *mashua* as an economically important value-added crop. This species is identified as a primary resource in the transition to a sustainable food bioeconomy based on the valorization of native Andean crops [7,32,64].

The future of *mashua* as a strategic resource will depend on the integration of fundamental science (genetics, physiology, and biochemistry) with technological application (processing and product formulation) and clinical validation. Methodological standardization is urgently required for reliable comparisons. Interdisciplinary research should also link biotechnology advances to concrete agro-industrial solutions. Furthermore, it is very important to enhance the linkage between science, markets, and producing communities where *mashua* is cultivated, ensuring that its valorization benefits not only scientific development but also high-Andean populations. This kind of comprehensive approach can transform *mashua* from a traditionally underused crop to an integral part of the global agenda on functional and sustainable foods.

## 8. Conclusions

*Mashua* is an Andean tuber with notable nutritional value and broad genetic, morphological, and ecophysiological diversity that enables it to adapt to extreme environmental conditions, making it a key crop for resilient agriculture in high-altitude Andean zones. Its tubers contain proteins, vitamin C, carotenoids, polyphenols, and glucosinolates with antioxidant, anti-inflammatory, antimicrobial, and antiproliferative properties, as demonstrated in in vitro studies and animal models.

Nevertheless, it has important limitations. Its functional compounds are not unique, and other conventional plant sources contain them in higher concentrations or with better clinical support. Glucosinolates are a double-edged sword: while they represent its main functional advantage, treatments to reduce bitterness or antinutritional effects inevitably compromise its bioactive potential.

The most significant gap separating *mashua* from functional ingredients with proven efficacy is the absence of clinical studies in humans. Although freeze-drying, microencapsulation, and extrusion technologies have improved bioactive preservation, challenges persist in sensory acceptance, standardization, scalability, and variability among accessions.

Therefore, the most realistic value proposition for *mashua* lies in adaptive Andean agriculture, biodiversity conservation and specialized markets, not in competing with global functional ingredients. Its commercialization requires comparative studies, clinical trials, techno-economic analyses and sensory optimization that address the obstacles limiting its global competitiveness.

Future research must prioritize comparative studies, human clinical trials, process optimization that balances bioactivity and palatability, and realistic techno-economic assessments to establish evidence-based applications that benefit both scientific advancement and Andean agricultural communities.

## Figures and Tables

**Table 1:** Proximal composition of *the mashua* (dry basis).

Component	Reported Range (d.b)	Unit	Source
Protein	6.96–18.25	g/100 g	[10,12,29,30,39,40]
Total carbohydrates	73.79–85.8	g/100 g	[10,30]
Total sugars	27.70–53.5	g/100 g	[29,39,41]
Lipids	0.92–1.67	g/100 g	[10,29,32,39]
Dietary fiber	0.7–15.59	g/100 g	[10,39]
Energy	343.66–440	kJ/100 g	[12,26,32,42]

**Table 2:** Micronutrient content of *mashua* (dry basis).

Compound	Reported Range (d.b.)	Unit	Source
Vitamin C	0.65–446	mg/100 g	[17,23,30]
Total carotenoids	0.1–8.6	mg/100 g	[17,23,29]
*β*-carotene (Provit. A)	1.39–9.67	mg/100 g	[34]
Calcium (Ca)	50–90	mg/100 g	[29,34]
Potassium (K)	990–3250	mg/100 g	[12,29,30,34]
Phosphorus (P)	420–730	mg/100 g	[12,34]
Zinc (Zn)	2.95	mg/100 g	[34]

**Table 3:** Bioactive compounds in *mashua*.

Compound	Reported Range (d.b.)	Unit	Source
Total glucosinolates	27–9660	µmol/100 g	[17,21,22,23,34]
Total polyphenols	116–1630	mg GAE/100 g	[18,23,30]
Caffeic acid	≈888 (as glycoside)	mg/100 g	[33]
Total anthocyanins	13–148,901	mg C3G/100 g	[18,30]
Rutin	144	mg/100 g	[33]
Quercetin	0.11–7.6	mg/100 g	[16,48]

**Table 4:** *Mashua* bioactive compounds and functional properties.

Biological Activity	Active Compounds	Main Results	Variety/Extraction	Source
Antioxidant	Proanthocyanidins, flavan-3-ols, and anthocyanins	LDL inhibition: metal-catalyzed stability (Cu^2+^) extended by flavan-3-ol-rich fractions Anthocyanins retain activity despite limited bioaccessibility and moderately inhibit carbohydrate digestion.	Purple accessions: purified extracts	[33,42,46,51]
Anti-glycation (AGEs)	Macamides (S-N-(α-methylbenzyl)-oleamide/linoleamide)	BSA–MGO inhibition: 3.39–8.53%. AGE degradation: 6.58–18.08%. → Specific activity not described in the narrative and relevant to metabolic processes.	Isolated compounds	[50]
Anti-inflammatory	Alkamides, macamides, and anthocyanins	Potent activation of Nrf2 by synthetic analog (0.03 nM). NF-κB inhibitory activity documented in topical extracts (histological support).	Ethanolic extracts and synthetic compounds	[35,38,52,53]
Antiproliferative/cytotoxic	Isothiocyanates derived from benzyl glucosinolate	Reversible spermatotoxic effects linked to isothiocyanate covalent reactivity (3.7 g/100 g). → Distinctive result of *mashua* compared with other tubers.	Aqueous extracts	[5,54,55,56]
Neuroprotective	N-benzyl linoleamide and analogs	Nrf2 activation with EC_50_ of 15.95–21.7 nM The most reactive analog activates Nrf2 at 0.03 nM (non-redundant due to extraordinary potency).	Synthetic and natural derivatives	[57]
Cardioprotective/hepatoprotective	*β*-sitosterol, phytosterols, triterpenoids, ethyl acetate fraction	EaF: high antioxidant capacity 200.2 µmol TE/mL and 22.2 mg GAE/mL (key metric for hepatoprotection). Evidence of reduced angiogenesis (not detailed in the narrative).	Lipophilic extracts and EaF	[36,58]

Note: BSA-MGO, bovine serum albumin-methylglyoxal; AGE, advanced glycation end products; TE, Trolox equivalents.

**Table 5:** Optimized processing technologies and their impact on the functional properties of *mashua*.

Technology	Application	Optimized Parameters	Main Outcome/Functional Benefit	Source
Ultrasound-Assisted Extraction	Anthocyanin extraction	48 °C, 33.93% ethanol, 20 min	Maximum yield of anthocyanins and polyphenols; high antioxidant capacity.	[49]
Microencapsulation (Spray-drying)	Stabilization of the extracts	160 °C inlet temperature, 2% OSA starch	High encapsulation efficiency, low hygroscopicity, and low water activity.	[10]
Freeze-Drying	Dehydration/Flour	Low temperature/vacuum	Retention of >80% vitamin C; preservation of glucosinolate profile	[24]
Extrusion	Functional Snacks (2G/3G)	20% flour substitution	Increased dietary fiber and phenolic content; minimal nutritional degradation.	[74]
3D Printing	Personalized Food	Pretreatment with cooked puree	Optimal viscoelasticity (G′ > G″) and shape fidelity due to starch retrogradation.	[9]

Note: OSA, octenyl succinic anhydride; 2G, second-generation snacks; 3G, third-generation snacks; G′, storage modulus (elastic component); G″, loss modulus (viscous component).

**Table 6:** Commercial perspectives and sustainability pathways for *mashua* across various industries.

Commercial Sector	Market Opportunities	Limiting Barriers	Viability Indicators	Implementation Strategies	Source
Premium Functional Ingredients	Segmentation of purple is yellow varieties; R^2^ = 0.7 color–phenolic correlation	Inadequate regulatory frameworks for nutraceuticals	Tt-23: 220.83 ± 0.42 mg GAE/100 g; variability, 2.73–6.825 mg/g d.b.	Geographical origin and industrial visual classification	[16,26,30,33]
Natural industrial colorants	Synthetic dye substitution; stability of 3D printing; cosmetology applications	Competition with established dyes and lack of commercialization	Validated dimensional stability; stable and visually appealing natural pigments	Classification of Exotic, Organic, and Natural Products as Consumption Incentives	[74,79]
Mass of processed foods consumed	Snacks (71–84% porosity), flours (>80% acceptance), beverages (pH 3.9, >8 log CFU/mL, +38% antioxidant activity)	Consumer education, limited distribution, and uncommon organoleptic properties	Compliance with INEN (<40% fat); 60-day stability; wine 9.5% *v*/*v* alcohol; yogurt +6% higher acceptability	Partial substitution in established and differentiated formulations	[25,26,49,74,75,77]
Biodegradable packaging	Sustainable plastics alternative; high transparency; synthetic additive replacement	Scaling of starch extraction and production costs	Satisfactory barrier properties and adequate thermal stability	Integration into SSCs and technological partnerships	[80,81]
Extractive technologies	UAE: 251 mg GAE/100 g, DPPH >85%; superior time–energy efficiency	Protocol standardization, specialized purification, and methodological heterogeneity	Freeze-drying: >60-day bioactivity; 35–80% efficiency, >75% retention	Industrial technology transfer and site-specific optimization	[10,49,75]
Animal feed	25% *mashua* supplementation improves productivity; excellent feed for cooked pigs	Undervalued feed rations; traditional management increases costs	Significant effect on carcass fat content (*p* < 0.05); *O. tuberosa* leaves as bovine forage	Replacement of soy and maize due to their high nutritional value and exploitation of their nutritional properties	[79,82]
Germplasm conservation	SIT-RITA^®^ micropropagation, genetic diversity conservation, and scalable production	Expansion of germplasm banks and limited exchange of protocols	Virus-free microtubers; efficient seed systems; studied 27 morphotypes	Regional cooperation among Andean countries; development of appropriate technologies; availability of germplasm	[7,32,64,83]
International markets	Peruvian scientific leadership (48 documents, 1047 citations); strategic collaborations; New Zealand demand for scientific leadership	Limited availability, regulatory entry barriers, and lack of awareness among consumers	Perú–Belgium–Spain–Brazil partnerships; available germplasm; genetic improvement of species	Research–development–commercialization networks, clinical validation, and strategic provincial alliances	[64,79,84]

Note: GAE, gallic acid equivalents; d.b., dry basis; INEN, Ecuadorian Standardization Service; UAE, ultrasound-assisted extraction; DPPH, 2,2-diphenyl-1-picrylhydrazyl; CFU, colony-forming units; *O. tuberosa*, *Oxalis tuberosa*; SIT-RITA^®^, Temporary Immersion System (Sistema de Inmersión Temporal—Recipient for Immersion Temporary Automated).

**Table 7:** Main Challenges and Future Research Lines in *Mashua*.

Identified Challenge	Evidence/Observed Limitation	Proposed Research Line
Variability in Results Reporting	Values reported on fresh vs. d.b.; non-standard units	Analytical protocols on a d.b. using SI units
Methodological heterogeneity in bioactive compound determination	Different techniques (spectrophotometry vs. HPLC) yield poorly comparable results	Harmonized methodologies and inter-laboratory validation
Lack of clinical trials	Evidence Limited to In Vitro and Animal Models	Human clinical studies on bioavailability and safety
Scarce postharvest information	Limited evidence on the stability of bioactive compounds during storage and transport	Kinetics of vitamin C, anthocyanins, and glucosinolate degradation
Poorly scaled emerging processes	Technologies, such as microencapsulation and 3D printing, are tested only at the laboratory level.	Validation at pilot and industrial scales with cost-effectiveness assessment
Low sensory acceptance	Bitterness and astringency associated with glucosinolate use	Applying partial detoxification techniques and blending with other ingredients
Insufficient integration into value chains	Limited articulation among Andean producers, industry, and the global market	Designing inclusive business models with traceability and certification
Poorly documented environmental impact	Lack of life cycle analysis in cultivation and processing	Development of studies on sustainability and carbon footprint

Note: d.b., dry basis; HPLC, high-performance liquid chromatography; SI, International System of Units. Information obtained from [5,7,23,24,31,35,38,52,56,73,75,80,81,83,84,85,86,87].

## Data Availability

No new data were created or analyzed in this study.

## Abbreviations

The following abbreviations are used in this manuscript:

ABTS	2,2′-Azino-bis(3-ethylbenzothiazoline-6-sulfonic acid)
AFLP	Amplified Fragment Length Polymorphism
AGE	Advanced Glycation End Products
APC	Antioxidant Power Composite Index
aPTT	Activated Partial Thromboplastin Time
BSA-MGO	Bovine Serum Albumin–Methylglyoxal
C3G	Cyanidin-3-Glucoside Equivalents
CFU	Colony-Forming Units
CI	Combination Index
d.b.	D.b.
DPPH	2,2-Diphenyl-1-picrylhydrazyl
EaF	Ethyl Acetate Fraction
5-FU	5-Fluorouracil
FRAP	Ferric Reducing Antioxidant Power
GAE	Gallic Acid Equivalents
GSL	Glucosinolates
HPLC	High-Performance Liquid Chromatography
HPLC-DAD/MS	High-Performance Liquid Chromatography with Diode Array Detector and Mass Spectrometry
ISSR	Inter-Simple Sequence Repeats
LDH	Lactate Dehydrogenase
LDL	Low-Density Lipoproteins
LPS	Lipopolysaccharide
MTT	3-(4,5-Dimethylthiazol-2-yl)-2,5-Diphenyltetrazolium Bromide
NF-κB	Nuclear Factor Kappa B
Nrf2	Nuclear Factor Erythroid 2–Related Factor 2
ORAC	Oxygen Radical Absorbance Capacity
PT	Prothrombin Time
RAPD	Random Amplified Polymorphic DNA
RE	Retinol Equivalents
ROS	Reactive Oxygen Species
SSR	Simple Sequence Repeats
TBARS	Thiobarbituric Acid Reactive Substances
TE	Trolox Equivalents
TEAC	Trolox Equivalent Antioxidant Capacity
TNF-α	Tumor Necrosis Factor Alpha
